# Comfortable Meals in an Institution—Supper

**Published:** 1912-08-17

**Authors:** 


### INSTITUTIONAL HOUSEKEEPING.

### Comfortable Meals in an Institution—Supper.

To the enlightened mind, supper or any heavy meal taken late in the evening is a mistake. We know that a comfortable night depends upon our being free from the labour of digestion during sleep, and those only are the invariably cheerful members of society who content the demands of appetite not later than 7.30 o'clock, and retire for the night to rest sweetly and not to do the work that can best be accomplished with the aid of exercise. But as there must be in most institutions long hours of evening toil a late evening meal is customary and acceptable to all, and we dare not find fault with what is necessary. Tired out for the most part, the nurses hear with relief the bell for supper, and hasten to assemble for the last pleasure of the day, for to many there is a particular fascination in a supper.

Some few there are, an increasing number, of simple habits and moderate requirements, who prefer to regulate their diet on principles of strict hygiene, and for these a cup of hot cocoa or milk with bread and cheese or butter suffice. But there are robust creatures, with strong digestions, whose appetites are keen, and who would manifest open discontent unless meat in some form were provided. Between these two classes are many others who would fain find some variation in their evening repast from other meals provided, and come to the table expectantly in search of appetising tit-bits. We all have seen in cookery books the remark that needs no explanation, "This makes an excellent supper-dish." It is something out of the common, which we should never dream of producing at breakfast, dinner, or tea; it was pre-eminently devised and invented for supper only. Who does not know the curious compounds that are eaten in cold-blood in the late evening hour by many who revel in "supper-dishes"? We may shudder and pass them by, but why judge our neighbours whose digestion is superior to our own? Let them have the opportunity of being happy in their own way, it will not hurt us. The matron who desires to satisfy all alike will not be tied and bound to her own tastes and ideas, and she is not responsible for those of her household beyond the duty of gratifying them. Let her sit serene at the head of the table and dispense delicious Scotch broth or soup made out of bones and pieces, macaroni cheese, rice-pudding and stewed prunes, figs and sago, fish pie, kedgaree, remains of pudding or pie left from dinner—whatever she considers suitable and wholesome for her numerous family. But at the same time let her not forget to indulge occasionally the weakness of some who find an absolute relief from care in consuming cold mackerel in vinegar, pickled salmon or lobster, cheese and cucumber, cold sausage, pork-pie, pickled eggs, or anything else that may be poisonous and abominable to herself. She will find it does not hurt them in the least; far from it. On the other hand, there will arise in that happy establishment a spirit of good-humoured comradeship produced wherever the workers feel they are cared for individually and not lumped together to save trouble. A "cold supper," without the relief even of hot cocoa and milk, should never be offered to hard-working folk, save in the height of summer, and it is rare to find kitchen arrangements so primitive that "warming up" at least cannot be managed. It is positively wearisome to be summoned every night to consume even the handsomest joints of cold beef or mutton, with the alternative of bread and cheese. The supper ought to be a pleasant informal meal, when no one will need to eat a great deal, but all will be grateful to find their morsel tasty. Porridge has been found a suitable dish to present at this last meal, and many will be content to retire to rest on this and no more, with treacle or sugar or salt added to it. It is not always convenient to make porridge for a large party in the early morning, but it is quite as wholesome when taken at night, and can be varied in many ways; perhaps the most appetising is Quaker Oats made with half milk.

### Housekeeping Queries.

#### Supper Dishes with Eggs.

"HOUSEKEEPER" *writes: "Could you give me a few ideas for cooking eggs? Some patients in our home are obliged to have them daily, and they are tired of my few recipes."*

SCRAMBLED EGGS WITH RICE is always popular. Beat well a couple of new-laid eggs. Melt an ounce of butter in a small pan, add to it two small tablespoonfuls of well-boiled rice. Heat the latter well in the butter, then add one tablespoonful of milk, the beaten eggs, and a careful seasoning of salt and pepper. Stir the contents of the pan over a gentle heat until all becomes a soft, creamy mass. Be careful not to overheat the eggs or they will become tough and hard, and consequently indigestible. Heap the mixture neatly on rounds of hot, buttered toast, and pour round a little brown or tomato sauce, or, if preferred, serve plain.

SHIRRED EGGS.—Butter the inside of some paper ramakin cases or fireproof pipkins just large enough to hold an egg. Sprinkle the butter with a few browned crumbs. Break an egg carefully into each case, trying not to break the yolk, put a teaspoonful of milk, a tiny bit of butter, and a dust of salt and pepper on each egg. Stand the cases in the oven, or in a saucepan on the stove, adding enough boiling water to come half way up the cases before putting the lid on the pan, and bake or steam gently until lightly set. Then serve in the cases quickly.

EGG AND TOMATO CUSTARDS are good and quite a change. Beat two eggs together, add two tablespoonfuls of tomato pulp—that is, the pulp of ripe tomatoes rubbed through a sieve or strainer. Mix with these two tablespoonfuls of milk or cream and seasoning. Butter some small cups or cases, pour in the custard, and bake slowly for about ten minutes, or until lightly set. Remember if custard is cooked quickly it will be honeycombed through and separate. Two teaspoonfuls of grated cheese may be added to the above mixture, if desired.

